# Preserved Ratio Impaired Spirometry (PRISm) from an Epidemiological Perspective

**DOI:** 10.3390/jcm14217831

**Published:** 2025-11-04

**Authors:** Beate Stubbe, Till Ittermann, Anne Obst, Henry Völzke, Ralf Ewert

**Affiliations:** 1 Department of Pneumology, Internal Medicine B, University Medicine Greifswald, 17475 Greifswald, Germany; 2 Department of SHIP/Clinical-Epidemiological Research, Institute for Community Medicine, University Medicine Greifswald, 17475 Greifswald, Germany

**Keywords:** spirometry, lung function, dyspnea, cardiopulmonary exercise testing, mortality

## Abstract

**Background:** The term preserved ratio impaired spirometry (PRISm) is defined as post-bronchodilator forced expiratory volume in 1 s (FEV_1_) <80% predicted and FEV_1_/forced vital capacity (FVC) ratio ≥0.7 or ≥lower limit of normal (LLN). The population prevalence is estimated to be between 3% and 20%. PRISm does not indicate a specific lung disease but is associated with functional limitations, respiratory symptoms, comorbidities, and mortality. The aim of this study is to analyze the PRISm prevalence in an excellently characterized epidemiological study, to obtain better insight into the influence of comorbidities on PRISm development and its impact on overall mortality. **Methods:** We included 3403 healthy subjects from the Study of Health in Pomerania (SHIP) and 507 individuals with PRISm. Data from lung function testing, cardiopulmonary exercise testing (CPET), and echocardiography were compared in both groups. Comorbidities, as well as cardiovascular and all-cause mortality data, were analyzed. **Results:** Individuals in the PRISm group reported more often a history of myocardial infarction, hypertension, type 2 diabetes, dyspnea, and lung disease, and had more unfavorable median values for most of the lung function, CPET, and echocardiographic parameters compared to the non-PRISm group. Furthermore, they were older, more often current smokers, and had higher body fat marker values. Likewise, all-cause and cardiovascular death were more frequently observed in the PRISm group. **Conclusions:** Future studies are warranted to identify the underlying mechanisms and longitudinal progression of PRISm. However, our findings reveal that PRISm is not only associated with cardiovascular comorbidities but also with increased dyspnea, an impaired exercise capacity, and mortality.

## 1. Introduction

Lung function can be considered as a marker of general health and survival [1]. The GOLD 2025 report states that “Pre-Chronic Obstructive Pulmonary Disease (Pre-COPD)” can be found in individuals who complain of respiratory symptoms and/or have structural lung abnormalities and/or physiological dysregulations without the presence of airflow obstruction, identified by a forced expiratory volume in one second (FEV1) to forced vital capacity (FVC) ratio exceeding 0.70 in post-inhalation bronchial spirometry. “PRISm (preserved ratio impaired spirometry)”, a subtype of Pre-COPD, is defined by a FEV1/FVC ratio of ≥0.7 post-bronchodilation, together with a FEV1 that is less than 80% of the predicted value [2,3,4]. This condition has been defined as Global Initiative for COPD unclassified (GOLD-U) [2], restrictive [5,6], or nonspecific pattern [7].

A previous epidemiological study reveals an overall prevalence of 5%, while among ever smokers the prevalence was 10% [8]. Other studies reported a population prevalence of PRISm of between 3% and 20% [9,10]. Even though PRISm does not indicate a specific lung disease, it is associated with respiratory symptoms, functional limitations, comorbidities, and mortality [4,11,12]. PRISm has an increased 5-year mortality and morbidity compared to those with normal spirometry [11]. A possible progression from PRISm to chronic obstructive pulmonary disease (COPD) has been described in cohort studies in 25% of the patients in the cohort after 5 years [11] and 32% of the patients in the cohort after 4.5 years [10]. Therefore, PRISm can be considered as pre-COPD [13]. Individuals with PRISm represent a heterogenous population with a wide range of lung function impairment and radiographic emphysema [14]. Smoking exposure, high body mass index (BMI), and reduced total lung capacity (TLC) have also been associated with PRISm [2]. Smokers with prominent airway disease commonly progress from normal spirometry to PRISm and then to COPD [8]. However, it remains unclear how to apply the term PRISm to nonsmokers [15].

The aim of this study was (1) to analyze PRISm prevalence in an excellently characterized epidemiological study, (2) to obtain better insight into the influence of comorbidities on PRISm development, and (3) to investigate PRISm’s impact on mortality.

## 2. Materials and Methods

### 2.1. Participants

The study population comprised participants from the Study of Health in Pomerania (SHIP). The SHIP project is a population-based epidemiological assignment run by the Community Medicine Research Network of the University Medicine Greifswald, collecting data on multiple health outcomes from the general adult population of Pomerania, northeast Germany. Study details are provided elsewhere [16,17].

From the 6753 individuals who participated in SHIP-START-2 or SHIP-TREND-0 we excluded 2843 individuals for not attending basic and advanced pulmonary function tests (PFTs) and incremental cardiopulmonary exercise testing (CPET), resulting in a study population of 3910 individuals (49.6% women). The study was conducted according to the guidelines of the Declaration of Helsinki and approved by the Institutional Ethics Committee of the University of Greifswald (SHIP-2/SHIP-TREND/BB39/08, date of approval 19.06.2008). Informed consent was obtained from all subjects involved in the study.

### 2.2. Lung Function Testing

Lung function examinations were conducted using equipment produced by Jaeger, Hoechberg, Germany, that meets the American Thoracic Society (ATS) and European Respiratory Society (ERS) criteria and recommendations [18]. The volume signal of the equipment was calibrated with a 3.0 L syringe connected to the pneumotachograph in accordance with the manufacturers’ recommendations and at least once on each day of testing. Barometric pressure, temperature, and relative humidity were registered every morning. Calibration was examined under ambient temperature and pressure conditions (ATP) and the integrated volumes were BTPS (Body Temperature Pressure Saturated)-corrected.

The participants performed at least three forced expiratory lung function maneuvers in order to obtain a minimum of two acceptable and reproducible values. Immediate on-screen error codes indicated the major acceptability (including start, duration, and end of test) and reproducibility criteria. The procedure was continuously monitored by a physician. Before the tests, the required maneuvers were demonstrated by the operator, and the individuals were encouraged and supervised throughout the performance of tests. Lung function variables were measured continuously throughout the baseline breathing and the forced maneuvers. Spirometry flow volume loops were conducted in accordance with ATS recommendations: in a sitting position while wearing nose clips.

The best results for FVC and FEV_1_ were taken. The ratio of FEV_1_ to FVC (expressed as a percentage) was calculated from the largest FEV_1_ and FVC.

### 2.3. Cardiopulmonary Exercise Testing

A symptom-limited CPET employing a calibrated electromagnetically braked cycle ergometer with seat height adjustment (Ergoselect 100; Ergoline, Bitz, Germany) was performed according to a protocol modified from Jones et al. [19]. The protocol started with 3 min resting, 1 min unloaded cycling, and increasing workload in a stepwise manner by 16 watts × min^−1^, aiming for limiting symptoms in 8–12 min. The CPET metabolic and ventilatory measurements were averaged as 10 s bins (MasterScreen™ CPX, CareFusion^®^, Höchberg, Germany). Based on the visual inspection of minute ventilation (V’_E_)/O_2_ uptake (V’O_2_) and V’_E_/CO_2_ output (V’CO_2_) and time-aligned end-tidal partial pressure for O_2_ and CO_2_ (*P*_ET_O_2_ and *P*_ET_CO_2_, respectively) during the exercise, a certified technician identified the emergence of the “first” ventilatory threshold (VT_1_) [20].

### 2.4. Echocardiography

Two-dimensional, M-Mode, and Doppler echocardiography were performed using the Vivid-I system (GE Medical Systems, Waukesha, WI, USA) as described in detail elsewhere [16]. Measurements of the LV end-diastolic diameter (LVD, in cm), interventricular septal thickness (IVSD, in cm), and the posterior wall thickness (PWD, in cm) were performed according to the guidelines of the American Society of Echocardiography [21,22]. Left ventricular mass (LVM, in g) was determined according to the formula LVM = 0.8 × 1.04 × [(LVD + IVSD + PWD)^3^ − LVD^3^)] + 0.6, as described by Devereux et al. [23]. LVM was indexed to body height to the allometric power of 2.7 (LVMI = LVM/(height (m))^2.7^) [24].

### 2.5. Magnet Resonance Imaging (MRI)

Whole-body MRI has been performed in the studies since 2008 using a 1.5-T MR (magnetic resonance) imager (Magnetom Avanto, Siemens Medical Systems, Erlangen, Germany) [25]. Contrast-enhanced cardiac MRI and MR angiography have been performed in men, whereas cardiac MRI and MR mammography have been performed in women in the first examinations (SHIP-START-2 and SHIP-TREND-0) and the first follow-ups (SHIP-START-3 and SHIP-TREND-1). Over the examination waves, only minor changes to the applied sequences were made.

### 2.6. Statistical Methods

The characteristics of the study population are stratified by PRISm status as absolute numbers and percentages for categorical data and as median, 25th percentile, and 75th percentile for continuous data. Associations of behavioral risk factors with PRISm status were calculated using logistic regression models adjusted for age, sex, smoking status, and body mass index. Associations were also calculated, stratified by sex. Furthermore, PRISm status was associated with prevalent diseases by logistic regression models using the same adjustment set in the whole population as well as in men and women separately.

Multivariable Cox regression models were used to associate PRISm status with all-cause and cardiovascular mortality. Multivariable linear regressions were used to associate PRISm status with markers of lung function, CPET, and echocardiography. A *p* < 0.05 was considered as statistically significant. All analyses were carried out with STATA 18.5 (Stata Corporation, College Station, TX, USA).

## 3. Results

Overall, the PRISm group contained 507 individuals (13.0%) with a median age of 60 years (Table 1). Compared to the non-PRISm group, individuals in the PRISm group were older, more often current smokers, and had higher fat marker values. Furthermore, individuals in the PRISm group reported more often a history of myocardial infarction, hypertension, type 2 diabetes mellitus, dyspnea, and lung disease. Individuals in the PRISm group had more unfavorable median values for most of the lung function, CPET, and echocardiographic parameters compared to the non-PRISm group. Likewise, all-cause and cardiovascular death were more frequently observed in the PRISm group.

After adjustment for age, sex, and BMI, current smokers had a higher chance of being in the PRISM group compared to never smokers (Table 2). This effect was stronger in men than in women. BMI, waist circumference, and fat mass were positively associated with PRISm status. Again, these associations were stronger in men than in women. We observed no significant association between fat-free mass and PRISm status.

Compared to individuals in the non-PRISm group, individuals in the PRISm group had a higher chance of myocardial infarction, type 2 diabetes mellitus, dyspnea at moderate or heavy load, and lung disease, while there was no significant association of PRISm status with stroke, atrial fibrillation, and arterial hypertension (Table 3). In men, PRISm status was significantly associated with myocardial infarction, dyspnea, and lung disease, whereas in women, PRISm status was associated with type 2 diabetes mellitus and lung disease. In the total population and in women but not men, PRISm status was associated with all-cause mortality, while there was no significant association with cardiovascular mortality.

Except for the carbon monoxide transfer coefficient, PRISm status was significantly associated with all lung function parameters (Table 4). For many parameters, including maximal expiratory flow, expiratory peak flow, residual volume, and DLCO, these associations were stronger in men than in women.

With two exceptions, individuals in the PRISm group had worse CPET values than individuals in the non-PRISm group (Table 5). Like for the lung function parameters, the observed associations were stronger in men than in women for many CPET parameters including V’O_2_ peak, V’E/V’CO_2_ VT_1_, V’E/V’CO_2_ slope, oxygen pulse peak, and tidal volume peak.

Regarding echocardiographic parameters, we only found an inverse association between PRISm status and the left atrium diameter (Table 6). This association was observed in the total population, but in separate analyses only in men but not in women. For cardiac MRI parameters (ejection fraction, myocardial mass, end systolic and end diastolic volume, stroke volume, and myocardial thickness) we did not find differences between the PRISm and the non-PRISm groups.

## 4. Discussion

PRISm is associated with significant morbidity, including increased respiratory symptoms [12,14], reduced exercise tolerance [14], and increased rates of respiratory-related hospitalizations and deaths [26] relative to normal spirometry. Despite the associations between PRISm and cardiovascular comorbidities being differently reported throughout the existing studies, a higher PRISm prevalence can be found in obese individuals and current smokers [27].

Our findings reveal that PRISm is not only associated with increased dyspnea but also with cardiovascular comorbidities, an impaired exercise capacity, and mortality. In addition, individuals in the PRISm group were older, more often current smokers, and had a higher BMI compared to the non-PRISm group. PRISm participants showed a statistically lower TLC and FVC than healthy controls. RV/TLC was significantly higher in the PRISm group than the controls.

These associations were stronger in men than in women. However, the existence of PRISm is a risk factor for mortality, despite adjustment for comorbidities and smoking. This supports that impaired lung function contributes independent information relevant to overall survival [4,6,12]. Regarding prognosis in general, PRISm shows considerable variation. It ranges from improvement in lung function to the development of COPD [27].

The prevalence of PRISm in population-based studies ranges from 7.1% to 20.3% [6,12,28,29]. The overall prevalence of PRISm in our cohort is similar to other population-based studies [5,30,31].

The relationship of PRISm with obesity and diabetes is incompletely understood. While increased total and abdominal adiposity has been associated with reduced FEV_1_ and FVC, as well as decreases in TLC [32], lung function values of obese subjects typically remain within the normal range; thus, the degree of impairment in lung function in PRISm subjects is unlikely to be due solely to increased body mass [32,33] and therefore does not account for the severity of the lung function impairment observed with PRISm. Interestingly, both high and low BMI are associated with an increased risk of PRISm [4]. Our data indicate that a higher BMI is likely to be associated with PRISm. From a clinical perspective, PRISm varies from ventilation–perfusion mismatches that are due to obesity and typically reversible, to severe often irreversible lung pathologies. Both are associated with an increase in hypoxemia. This underscores the importance of the underlying mechanisms and potential for reversibility in clinical assessments [34]. The Rotterdam Study reported different subsets of PRISm and emphasized the role of PRISm as a restrictive pattern associated with obesity and heart failure that had a remarkable impact on mortality [10]. Focusing on aspects of prevention, Marott et al. proposed that if PRISm is detected early enough, past episodes of PRISm may not influence long-term health outcomes if they are resolved in early adulthood [26]. Despite not meeting COPD criteria, these patients require careful observation because of their risk for COPD development and concomitant medical utilization.

Taking a closer look at identifying smokers and ex-smokers who met the PRISm criteria, we found that current smokers had a higher chance of being in the PRISm group than never smokers. Recent studies have found that smokers and ex-smokers who meet the PRISm criteria have poor clinical outcomes [10,35]. It may be that many current smokers in the PRISm group did not experience respiratory symptoms, did not visit hospitals, and were not warned to stop smoking. Current smokers in the PRISm group may develop COPD unless they stop smoking, as previously shown [36]. Physicians should check the smoking status of PRISm patients more carefully, and should strongly recommend that they stop smoking.

### Strengths and Limitations

The most significant strength of the study is a thoroughly phenotyped cohort with valid data on diverse comorbid and behavioral conditions and consistent results in subgroup analyses for fixed thresholds, as well as lower limit of normal criteria to define lung function and exercise impairment. The lack of post-bronchodilator spirometry may have resulted in an overestimation of PRISm, as this is not in line with the PRISm definition. Nevertheless, a couple of studies have shown that the prevalence of PRISm based on pre-bronchodilator spirometry is comparable to the incidence of PRISm reported using postbronchodilator spirometry [4,9,37]. The HUNT study found that mortality was better predicted by post-bronchodilator than by pre-bronchodilator spirometry [38]. The reported study population was, in general, middle-aged and elderly Caucasian individuals and might therefore not be applied to other ethnic groups and younger individuals.

## 5. Conclusions

We conclude that PRISm is a complex state of pulmonary function limitation, the course and prognosis of which vary individually. Subjects with PRISm should be carefully monitored for COPD development. Despite the association with COPD and SAD (small airway dysfunction), effective treatments are still lacking. This is what future research needs to focus on in order to improve long-term health outcomes for patients. Additional studies are needed to investigate the mechanism related to PRISm. The application of artificial intelligence, its role in predicting PRISm subtypes and modeling ventilation function, and its opportunities for imaging techniques seem to be promising.

## Figures and Tables

**Table 1:** Characteristics of the study population stratified by PRISm.

	PRISm Group		
	No	Yes	*p* *
**N**	3403 (87.0%)	507 (13.0%)	
Age (years)	53 (43; 64)	60 (50; 70)	<0.001
Sex			
Men	1700 (50.0%)	272 (53.6%)	0.121
Women	1703 (50.0%)	235 (46.4%)	
Smoking status			
Never	1333 (39.2%)	161 (31.8%)	<0.001
Former	1397 (41.1%)	205 (40.5%)	
Current	667 (19.6%)	140 (27.7%)	
Body height (cm)	170 (163; 177)	171 (164; 177)	0.787
Body weight (kg)	79 (69; 91)	83 (72; 95)	<0.001
Body mass index (kg/m^2^)	27.3 (24.6; 30.4)	28.8 (25.2; 32.0)	<0.001
Waist circumference (cm)	90 (80; 100)	96 (85; 105)	<0.001
Fat mass (kg)	21.9 (17.3; 27.7)	24.3 (18.6; 31.8)	<0.001
Fat-free mass (kg)	55.8 (47.1; 66.6)	57.0 (47.5; 67.2)	0.073
**History**			
Myocardial infarction	70 (2.1%)	29 (5.7%)	<0.001
Stroke	57 (1.7%)	12 (2.4%)	0.269
Atrial fibrillation	452 (13.3%)	73 (14.4%)	0.491
Cancer	239 (7.0%)	44 (8.7%)	0.173
Hypertension	1605 (47.3%)	305 (60.2%)	<0.001
Diagnosed type 2 diabetes	289 (8.5%)	84 (16.6%)	<0.001
Dyspnea			
No	2594 (76.4%)	277 (54.9%)	<0.001
@ heavy load	657 (19.3%)	171 (33.9%)	
@ moderate load	126 (3.7%)	51 (10.1%)	
@ low load	20 (0.6%)	6 (1.2%)	
Lung disease	147 (4.3%)	74 (14.8%)	<0.001
Drugs for obstructive airway diseases	87 (2.6%)	51 (10.1%)	<0.001
Estimated glomerular filtration rate (mL/min)	90 (76; 101)	85 (72; 97)	<0.001
**Lung function testing**			
Forced expiratory volume (FEV_1_) (% predicted)	99.6 (91.7; 107.5)	73.3 (66.7; 77.2)	<0.001
Forced vital capacity (FVC) (% predicted)	109.1 (97.6; 121.9)	85.4 (76.7; 97.0)	<0.001
FEV_1_/FVC (%)	80.3 (76.8; 83.3)	73.4 (67.8; 78.1)	<0.001
Maximal expiratory flow at 25% of FVC (L/s)	1.21 (0.87; 1.66)	0.55 (0.38; 0.80)	<0.001
Maximal expiratory flow at 50% of FVC (L/s)	3.93 (3.10; 4.87)	1.99 (1.44; 2.71)	<0.001
Maximal expiratory flow at 75% of FVC (L/s)	6.08 (4.95; 7.53)	4.04 (3.09; 5.05)	<0.001
Expiratory peak flow (L/s)	6.74 (5.37; 8.27)	4.96 (3.91; 6.12)	<0.001
Total airway resistance (kPa/L/s)	0.19 (0.14; 0.24)	0.28 (0.20; 0.38)	<0.001
Residual volume (RV) (% predicted)	102.1 (80.7; 121.6)	112.2 (91.9; 138.0)	<0.001
Total lung capacity (TLC) (% predicted)	103.2 (94.0; 112.2)	95.0 (84.6; 105.7)	<0.001
RV/TLC (%)	0.35 (0.28; 0.41)	0.46 (0.38; 0.52)	<0.001
Diffusing capacity for carbon monoxide (DLCO) (mL/min/kPa)	7.40 (6.30; 8.91)	6.38 (5.27; 7.52)	<0.001
DLCO (% predicted)	83.8 (75.9; 91.9)	73.5 (64.6; 83.8)	<0.001
Carbon monoxide transfer coefficient (Krogh index) (mmol CO/s × kPa)	1.37 (1.23; 1.49)	1.30 (1.15; 1.48)	<0.001
Krogh index (% predicted)	90.0 (81.4; 99.3)	89.9 (79.0; 101.0)	0.735
**Cardiopulmonary exercise testing (CPET)**			
Reason for termination of CPET			
Muscular fatigue	2432 (71.5%)	342 (67.5%)	0.089
Hip or knee pain	129 (3.8%)	17 (3.4%)	
Dyspnea	624 (18.3%)	117 (23.1%)	
Other	218 (6.4%)	31 (6.1%)	
Borg scale	8 (8; 9)	8 (8; 9)	0.192
Respiratory exchange rate @ peak	1.18 (1.11; 1.24)	1.13 (1.05; 1.20)	<0.001
Maximal workload (Watt)	148 (116; 196)	132 (100; 164)	<0.001
Maximal workload (% predicted)	117.0 (97.4; 146.8)	98.9 (81.3; 130.0)	<0.001
V’O_2_ peak (mL/min)	1890 (1500; 2400)	1646 (1307; 2062)	<0.001
V’O_2_ peak (mL/min/kg)	24.0 (19.7; 29.0)	20.1 (16.2; 24.5)	<0.001
V’O_2_ peak (% predicted)	96.0 (84.3; 108.2)	86.3 (73.1; 97.3)	<0.001
V’O_2_ VT_1_ (mL/min)	950 (800; 1200)	900 (750; 1100)	<0.001
V’_E_/V’CO_2_ VT_1_	27 (25; 30)	28 (25; 31)	<0.001
V’_E_/V’CO_2_ peak	30 (27; 33)	30 (27; 33)	0.313
V’_E_/V’CO_2_ slope	27 (25; 29)	28 (26; 31)	<0.001
*P*_ET_CO_2_ rest (mmHg)	32 (29; 35)	32 (29; 34)	0.529
*P*_ET_CO_2_ VT_1_ (mmHg)	39 (36; 43)	38 (34; 42)	<0.001
*P*_ET_CO_2_ VT_1_—*P*_ET_CO_2_rest (mmHg)	7 (5; 9)	6 (4; 8)	<0.001
Oxygen pulse peak (mL/beat)	12.7 (10.3; 15.5)	12.3 (10.0; 14.9)	0.016
Tidal volume peak (L/kg)	2.1 (1.8; 2.7)	1.8 (1.4; 2.2)	<0.001
Breathing frequency peak (breaths/min)	31 (27; 35)	32 (28; 36)	0.029
Ventilation (V’E) peak (L/min)	68 (53; 86)	58 (47; 71)	<0.001
V’E/maximal voluntary ventilation (%)	28 (22; 36)	33 (26; 42)	<0.001
Heart rate rest (bpm)	76 (68; 86)	76 (66; 85)	0.040
Heart rate peak (bpm)	157 (139; 171)	139 (118; 160)	<0.001
Heart rate peak—heart rate rest (bpm)	78 (62; 92)	62 (45; 79)	<0.001
Heart rate peak (% of the target heart rate)	94 (85; 100)	86 (76; 96)	<0.001
Alveolar–arterial gradient peak	29 (24; 36)	32 (26; 39)	<0.001
**Echocardiography**			
LV end-diastolic volume (mL)	112 (95; 131)	114 (97; 139)	0.041
LV end-diastolic volume index (mL/m^2.7^)	26.6 (22.9; 30.8)	27.5 (24.0; 31.9)	0.004
LV end-systolic volume (mL)	31.0 (23.6; 40.7)	32.5 (24.0; 43.6)	0.047
LV end-systolic volume index (mL/m^2.7^)	7.39 (5.72; 9.38)	7.65 (5.91; 10.14)	0.032
LV wall thickness (mm)	10.0 (8.9; 11.1)	10.1 (9.2; 11.4)	0.006
LV wall thickness index (mm/m^2.7^)	2.37 (2.09; 2.68)	2.47 (2.15; 2.79)	0.002
LV mass (g)	273 (225; 329)	292 (232; 350)	0.001
LV mass index (g/m^2.7^)	64.9 (55.2; 75.7)	68.4 (57.6; 83.2)	<0.001
LV stroke volume (mL)	78.9 (65.1; 94.8)	80.7 (66.0; 97.6)	0.251
LV stroke volume index (mL/m^2.7^)	18.8 (15.7; 22.4)	19.4 (15.7; 23.1)	0.125
LV ejection fraction (%)	71.8 (65.6; 77.4)	70.7 (63.7; 77.8)	0.205
E to E’ ratio	6.09 (5.12; 7.21)	6.37 (5.29; 7.72)	<0.001
Left atrium diameter (cm)	3.89 (3.54; 4.28)	4.02 (3.54; 4.38)	0.008
Left atrium diameter index (cm/m^2.7^)	0.93 (0.83; 1.05)	0.96 (0.84; 1.07)	0.010
**Mortality**			
All-cause mortality	222 (6.5%)	77 (15.2%)	<0.001
CV mortality	45 (1.3%)	20 (4.1%)	<0.001
Data are expressed as median, 25th percentile, and 75th percentile for continuous data or as absolute numbers and percentages for categorical data.			

* Wilcoxon test (continuous data) or χ^2^-test (categorical data).

**Table 2:** Associations of behavioral risk factors with the PRISm group.

	All Odds Ratio (95%-CI)	Men Odds Ratio (95%-CI)	Women Odds Ratio (95%-CI)
**Smoking status** former vs. never current vs. never	1.18 (0.94; 1.49) 2.63 (2.01; 3.44) *	1.42 (0.99; 2.04) 4.04 (2.70; 6.05) *	1.03 (0.74; 1.42) 1.72 (1.17; 2.52) *
**Body mass index (kg/m^2^)**	1.04 (1.02; 1.06) *	1.05 (1.02; 1.09) *	1.03 (0.99; 1.05)
**Waist circumference (cm)**	1.03 (1.02; 1.03) *	1.03 (1.02; 1.04) *	1.02 (1.01; 1.03) *
**Fat mass (kg)**	1.03 (1.02; 1.04) *	1.04 (1.03; 1.06) *	1.02 (1.01; 1.04) *
**Fat-free mass (kg)**	0.99 (0.97; 1.01)	0.99 (0.97; 1.01)	0.98 (0.95; 1.02)

Logistic regression models adjusted for age, sex (if not sex-specific), body mass index (for smoking), and smoking status (for anthropometric markers). Model for fat-free mass is further adjusted for fat mass. CI confidence interval. * *p* < 0.05.

**Table 3:** Associations of the PRISm group with prevalent diseases/events.

	All Odds Ratio (95%-CI)	Men Odds Ratio (95%-CI)	Women Odds Ratio (95%-CI)
**Myocardial infarction**	1.78 (1.10; 2.87) *	1.78 (1.06; 2.98) *	1.80 (0.53; 6.17)
**Stroke**	0.89 (0.46; 1.72)	1.19 (0.56; 2.53)	0.47 (0.11; 2.11)
**Atrial fibrillation**	1.06 (0.80; 1.39)	0.95 (0.66; 1.36)	1.20 (0.78; 1.85)
**Hypertension**	1.13 (0.90; 1.42)	1.04 (0.76; 1.42)	1.25 (0.89; 1.75)
**Diagnosed type 2 diabetes**	1.42 (1.06; 1.90) *	1.22 (0.83; 1.80)	1.72 (1.10; 2.67) *
**Dyspnea moderate or heavy load**	1.93 (1.37; 2.73) *	2.96 (1.77; 4.97) *	1.39 (0.85; 2.25)
**Lung disease**	3.50 (2.57; 4.76) *	3.26 (2.05; 5.17) *	3.67 (2.43; 5.56) *
	**Hazard ratio (95%-CI)**	**Hazard ratio (95%-CI)**	**Hazard ratio (95%-CI)**
**All-cause mortality**	1.39 (1.06; 1.81) *	1.20 (0.86; 1.68)	1.80 (1.14; 2.86) *
**Cardiovascular mortality**	1.64 (0.95; 2.83)	1.38 (0.74; 2.57)	2.70 (0.83; 8.72)

Logistic regression models adjusted for age, sex (if not sex-specific), body mass index, and smoking status. Mortality outcomes were analyzed by Cox regression adjusted for age, sex (if not sex-specific), body mass index, and smoking status. CI confidence interval * *p* < 0.05.

**Table 4:** Associations of the PRISm group with markers of lung function.

	All β (95%-CI)	Men β (95%-CI)	Women β (95%-CI)
**Maximal expiratory flow at 25% of FVC (L/s)**	−0.54 (−0.58; −0.50) *	−0.61 (−0.67; −0.54) *	−0.46 (−0.52; −0.41) *
**Maximal expiratory flow at 50% of FVC (L/s)**	−1.73 (−1.83; −1.63) *	−2.01 (−2.18; −1.84) *	−1.42 (−1.54; −1.30) *
**Maximal expiratory flow at 75% of FVC (L/s)**	−1.98 (−2.11; −1.84) *	−2.41 (−2.63; −2.19) *	−1.49 (−1.65; −1.34) *
**Expiratory peak flow (L/s)**	−1.66 (−1.81; −1.51) *	−2.02 (−2.26; −1.78) *	−1.25 (−1.43; −1.08) *
**Total airway resistance (kPa/L/s)**	0.10 (0.09; 0.11) *	0.09 (0.08; 0.11) *	0.11 (0.09; 0.12) *
**Residual volume (RV) (% predicted)**	13.4 (10.1; 16.8) *	14.5 (9.7; 19.2) *	12.0 (7.3; 16.7)
**Total lung capacity (TLC) (% predicted)**	−8.2 (−9.6; −6.7) *	−8.7 (−10.7; −6.7) *	−7.8 (−9.9; −5.6) *
**RV/TLC (%)**	0.08 (0.07; 0.09) *	0.08 (0.07; 0.09) *	0.07 (0.06; 0.09) *
**Diffusing capacity for carbon monoxide (DLCO) (mL/min/kPa)**	−0.82 (−0.94; −0.70) *	−1.04 (−1.23; −0.86) *	−0.53 (−0.67; −0.39) *
**DLCO (% predicted)**	−10.0 (−11.2; −8.8) *	−12.2 (−13.9; −10.4) *	−7.6 (−9.2; −5.9) *
**Carbon monoxide transfer coefficient (Krogh index) (mmol CO/s × kPa)**	−0.01 (−0.02; 0.01)	−0.02 (−0.04; 0.00)	0.01 (−0.01; 0.04)
**Krogh index (% predicted)**	−0.61 (−1.75; 0.54)	−1.92 (−3.64; −0.18) *	1.03 (−0.45; 2.51)

Linear regression models adjusted for age, sex (if not sex-specific), body mass index, and smoking status. CI confidence interval. * *p* < 0.05.

**Table 5:** Associations of the PRISm group with markers of cardiopulmonary exercise testing.

	All β (95%-CI)	Men β (95%-CI)	Women β (95%-CI)
**Maximal workload (Watt)**	−17.4 (−20.5; −14.3) *	−20.6 (−25.4; −15.9) *	−11.6 (−25.4; −15.9) *
**Maximal workload (% predicted)**	−14.8 (−17.2; −12.5) *	−12.9 (−15.3; −10.5) *	−15.9 (−19.9; −11.9) *
**V’O_2_ peak (mL/min)**	−168 (−207; −129) *	−207 (−269; −145) *	−100 (−143; −57) *
**V’O_2_ peak (mL/min/kg)**	−2.1 (−2.6; −1.6) *	−2.4 (−3.1; −1.7) *	−1.5 (−2.1; −0.9) *
**V’O_2_ peak (% predicted)**	−8.8 (−10.5; −7.1) *	−10.0 (−12.4; −7.6) *	−7.2 (−9.6; −4.7) *
**V’O_2_ VT_1_ (mL/min)**	−49 (−70; −27) *	−57 (−91; −23) *	−33 (−58; −8) *
**V’E/V’CO_2_ VT_1_**	0.93 (0.59; 1.29) *	1.17 (0.69; 1.67) *	0.55 (0.06; 1.04) *
**V’E/V’CO_2_ peak**	−0.24 (−0.68; 0.21)	−0.49 (1.13; 0.15)	−0.06 (−0.67; 0.55)
**V’E/V’CO_2_ slope**	0.80 (0.48; 1.13) *	0.88 (0.41; 1.34) *	0.62 (0.18; 1.06) *
***P*** **_ET_** **C** **O_2_ rest (mmHg)**	0.19 (−0.23; 0.62)	−0.06 (−0.64; 0.52)	0.56 (−0.07; 1.19)
***P*** **_ET_** **C** **O_2_ VT_1_ (mmHg)**	−0.34 (−0.88; 0.20)	−0.68 (−1.43; 0.06)	0.21 (−0.56; 0.98)
***P*** **_ET_** **C** **O_2_ VT_1_ —** ***P*** **_ET_** **C** **O_2_ rest (mmHg)**	−0.52 (−0.82; −0.22) *	−0.62 (−1.04; −0.19) *	−0.32 (−0.75; 0.11)
**Oxygen pulse peak (mL/beat)**	−0.54 (−0.78; −0.28) *	−0.76 (−1.16; −0.36) *	−0.19 (0.47; 0.09)
**Tidal volume peak (L/kg)**	−0.34 (−0.38; −0.30) *	−0.42 (−0.48; −0.35) *	−0.25 (−0.30; −0.20) *
**Breathing frequency peak (breaths/min)**	1.06 (0.35; 1.66) *	0.95 (0.13; 1.77) *	1.20 (0.32; 2.08) *
**Ventilation (V’E) peak (L/min)**	−8.8 (−10.4; −7.2) *	−10.7 (−13.3; −8.1) *	−6.1 (−7.9; −4.3) *
**V’E/maximal voluntary ventilation (%)**	7.0 (6.2; 7.7) *	8.6 (7.5; 9.8) *	5.3 (4.5; 6.1) *
**Heart rate rest (bpm)**	0.44 (−0.75; 1.64)	1.09 (−0.61; 2.79)	−0.49 (−2.17; 1.20)
**Heart rate peak (bpm)**	−8.0 (−9.8; −6.3) *	−8.2 (−10.7; −5.7) *	−7.6 (−10.2; −5.1) *
**Heart rate peak—heart rate rest (bpm)**	−8.5 (−10.2; −6.8)	−9.3 (−11.6; −6.9) *	−7.1 (−9.5; −4.8) *
**Heart rate peak (% of the target heart rate)**	−5.1 (−6.2; −4.0) *	−5.2 (−6.8; −3.7) *	−4.8 (−6.3; −3.2) *

Linear regression models adjusted for age, sex (if not sex-specific), body mass index, and smoking status. CI confidence interval. * *p* < 0.05.

**Table 6:** Associations of the PRISm group with echocardiographic parameters.

	All β (95%-CI)	Men β (95%-CI)	Women β (95%-CI)
**LV end-diastolic volume index (mL/m^2.7^)**	0.12 (−0.57; 0.82)	−0.30 (−1.31; 0.72)	0.53 (−0.41; 1.47)
**LV end-systolic volume index (mL/m^2.7^)**	0.26 (−0.11; 0.63)	0.08 (−0.50; 0.65)	0.41 (−0.06; 0.88)
**LV wall thickness index (mm/m^2.7^)**	−0.02 (−0.06; 0.03)	−0.02 (−0.08; 0.05)	0.01 (−0.05; 0.08)
**LV mass index (g/m^2.7^)**	0.71 (−0.96; 2.39)	−0.39 (−2.87; 2.10)	1.97 (−0.19; 4.13)
**LV stroke volume index (mL/m^2.7^)**	−0.13 (−0.73; 0.46)	−0.37 (−1.24; 0.49)	0.12 (−0.69; 0.92)
**LV ejection fraction (%)**	−0.76 (−1.92; 0.39)	−0.48 (−2.32; 1.36)	−0.92 (−2.37; 0.52)
**E to E’ ratio**	0.05 (−0.12; 0.22)	0.00 (−0.23; 0.24)	0.11 (−0.13; 0.36)
**Left atrium diameter index (cm/m^2.7^)**	−0.02 (−0.03; −0.01) *	−0.02 (−0.04; −0.01) *	−0.00 (−0.03; 0.02)

Linear regression models adjusted for age, sex (if not sex-specific), body mass index, and smoking status. CI confidence interval. * *p* < 0.05.

## Data Availability

Restrictions are imposed on the availability of data generated or analyzed during this study to ensure the preservation of patient confidentiality or due to the utilization of data under license. The application for data can be made following a standardized procedure: http://www2.medizin.uni-greifswald.de/cm/fv/ship/daten-beantragen/ (accessed on 9 September 2025).
